# Distinct Brain Areas involved in Anger versus Punishment during Social Interactions

**DOI:** 10.1038/s41598-018-28863-3

**Published:** 2018-07-12

**Authors:** Olga M. Klimecki, David Sander, Patrik Vuilleumier

**Affiliations:** 1 Swiss Centre for Affective Sciences, University of Geneva, Chemin des Mines 9, 1202 Geneva, Switzerland; 2 Laboratory for the Study of Emotion Elicitation and Expression, Department of Psychology, University of Geneva, Boulevard du Pont d’ Arve 40, 1205 Geneva, Switzerland; 3 Laboratory for Behavioral Neurology and Imaging of Cognition, Department of Neuroscience, Medical School, University of Geneva, rue Michel Servet 1, 1211 Geneva, Switzerland

## Abstract

Although anger and aggression can have wide-ranging consequences for social interactions, there is sparse knowledge as to which brain activations underlie the feelings of anger and the regulation of related punishment behaviors. To address these issues, we studied brain activity while participants played an economic interaction paradigm called Inequality Game (IG). The current study confirms that the IG elicits anger through the competitive behavior of an unfair (versus fair) other and promotes punishment behavior. Critically, when participants see the face of the unfair other, self-reported anger is parametrically related to activations in temporal areas and amygdala – regions typically associated with mentalizing and emotion processing, respectively. During anger provocation, activations in the dorsolateral prefrontal cortex, an area important for regulating emotions, predicted the inhibition of later punishment behavior. When participants subsequently engaged in behavioral decisions for the unfair versus fair other, increased activations were observed in regions involved in behavioral adjustment and social cognition, comprising posterior cingulate cortex, temporal cortex, and precuneus. These data point to a distinction of brain activations related to angry feelings and the control of subsequent behavioral choices. Furthermore, they show a contribution of prefrontal control mechanisms during anger provocation to the inhibition of later punishment.

## Introduction

Although anger and aggression have been researched since decades, there are still few studies on the neural functions that dissociate feelings of anger from the regulation of aggressive responses or reactive punishment behaviors. Anger and aggression are conceptually related, but anger does not always result in aggression^1,2^. In fact, the emotion of anger, which is defined as a negative emotional response to goal-blockage and unfair behavior by others^3,4^, is conceptually distinct from aggression, which is defined as an action intended to cause harm to another individual^5^. Although aggression is often perceived as maladaptive, certain forms of aggression may actually serve evolutionary adaptive purposes, such as securing resources or defending against attacks^6^.

The neural correlates of both anger and aggression have previously been investigated with computer-based paradigms such as the Taylor Aggression Paradigm^7,8^. In this competitive reaction time task, there is an alternation between provocation periods in which participants receive painful stimuli by another player and periods in which participants can retaliate by administering electric shocks to the other player. In this task, activations in amygdala and superior temporal sulcus (STS) when watching the opponent suffer were found to correlate with the intensity of the stimulus administered by the participant to the other player^7^. In a different study using this paradigm, activations in the anterior insula and the anterior cingulate cortex were related to reactive aggression^8^. However, in spite of the valuable insights obtained in these studies, there is so far no consensus about the brain activations associated with anger itself and the subsequent behavior toward anger-eliciting others. This may partly be due to the alternation of provocation and aggression inherent in the Taylor Aggression Paradigm, which makes it difficult to disentangle angry feelings from social behavior like punishment. Furthermore and as detailed below, most extant studies have focused on the neural correlates of either anger or aggression.

Pertaining to the brain activation patterns underlying anger, the majority of neuroimaging studies have investigated this emotion indirectly by showing angry faces^9,10^, or by using recall^11^, imagery^12,13^ or rumination^14^ of anger-eliciting situations. The results of these studies are very divergent in their findings. Whereas some findings point to an involvement of the orbitofrontal cortex^10^, other results suggest reduced activations in the orbitofrontal cortex and somatosensory cortex^11^ as well as increased activations in the anterior cingulate cortex (ACC) and insula^11^. Still other studies found an association of the temporal poles^12,13^ or the dorsal anterior cingulate cortex^14^ with anger.

The divergent nature of these results may have arisen from the indirect way of evoking anger, which may not be representative of actual feelings of anger. Paradigms in which participants are looking at angry faces, for instance, may simply activate the concept of anger^15^ or rather induce other emotions, such as fear. Moreover, studies employing paradigms relying on recall, imagery, or rumination rely heavily on the capacity of participants to internally relive a situation and this capacity may vary depending on the participants’ personality and the situation. In order to circumvent these shortcomings, it is important to measure neural activations while participants actually feel anger (for instance when seeing the face of a person who actually induced anger as opposed to seeing a face that merely expresses anger without necessarily inducing it in the viewer).

With regard to aggression, animal experiments and lesion studies in humans suggest that aggressive behavior is governed by subcortical brain circuits in the hypothalamus, amygdala, and brainstem, and that the frontal cortex, which integrates social information and can modulate activations in the hypothalamus and amygdala^16^. Whereas aggressive responses to a frustrating or threatening event are mediated by limbic systems, more controlled and goal-oriented aggression instead is regulated by higher order cortical systems^16^. In accordance with this assumption, evidence from lesion studies points to a causal role for the orbitofrontal and dorsolateral prefrontal cortex (DLPFC) in modulating the expression of aggressive behaviors^17^. Meta-analytic evidence also suggests that the DLPFC is an important brain region for the regulation of emotional responses^18^. Accordingly, it has been suggested that the DLPFC might be crucial for inhibiting aggression, as aggression may result as a consequence of deficits in emotion regulation^19^. Moreover, structural or functional impairments in the DLPFC, temporal lobe, and ACC have been observed in antisocial populations^20^. Although the above-mentioned lesion and neuroimaging studies on emotion regulation suggest that the DLPFC should play a role in aggression inhibition, functional neuroimaging data supporting this claim is so far missing.

**Figure 1:**
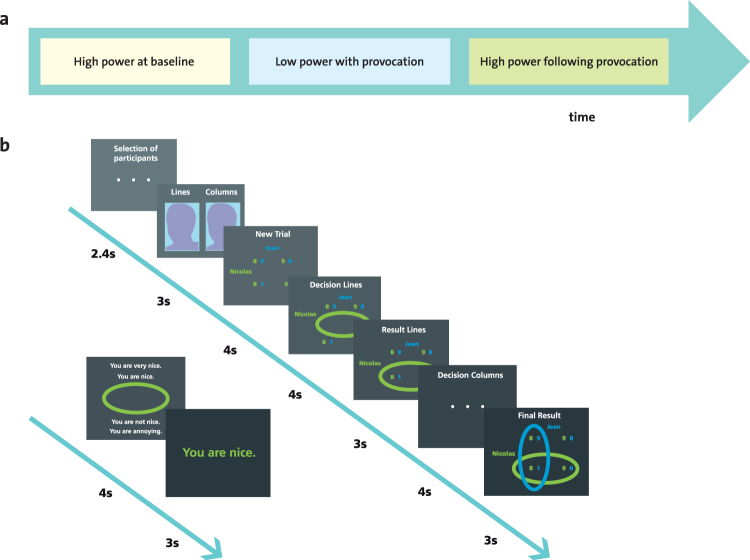
Study design. (**a**) The three phases of the Inequality Game (given in successive scanning runs). (**b**) Depiction of event frames of a single trial in the high power phase with their respective duration. Each time the participant is paired with one of the two other players, a screen is displayed that reads “Selection of participants”. Then, participants see a photograph of the *other* (fair or unfair) that is accompanied with the information that this player controls the columns in the high power phase (and the lines in the low power phase). This is followed by the display of the payoff matrix determining the distribution of gains for the two current players (“New Trial”), the decision screen for the person in high power (“Decision Lines”), the display of this decision (“Result Lines”), a display indicating that the other person is deciding (“Decision Columns”), and lastly the display of the other’s decision which serves at the final outcome display (“Final Result”). In the current example, the player with high power (here: Nicolas, in green) begins by selecting a line in the matrix, which is followed by the choice of the low power player (here: Jean, in blue) who selects a column. The intersection of their choice determines the final gain allocated to each player (here a competitive distribution is depicted in which Nicolas earns 8 CHF and Jean earns 1 CHF). Then, the player with high power can select one of four feedback messages for the other player.

In order to investigate brain activations related to angry feelings and the inhibition of related punishment behavior, we conducted an fMRI experiment with a final sample of 25 male participants who played the Inequality Game (IG), a novel paradigm recently introduced and validated in a previous study^2^. This interactive economic game (Fig. 1) has been shown to induce anger by confronting the participant with an unfair other player and to elicit different patterns of subsequent behavioral responses, including punishment of the unfair other, aggressive behavior towards the fair other as well as cooperative behavior towards both the fair and the unfair other^2^.

More specifically, in the IG, participants are iteratively paired with one of two other players (a fair and an unfair other) to engage in economic choices or feedback choices. A photograph of the other player is displayed each time the pairing changes. As illustrated in Fig. 1, the IG consists of three phases in which power is manipulated (high or low). The IG begins with a high power phase at baseline in which the participant is in control of choosing cooperative (i.e., high gain for both players) or competitive (i.e., high gain for participant and low gain for the other) economic outcomes for himself and one of the other players. In addition, the participant can decide whether to send nice (e.g., “You are very nice”) or derogatory (e.g., “You are annoying”) feedback messages to each of the two other players. This high power phase serves to assess participants’ behavioral preferences for engaging in cooperative or competitive behavior at baseline. It is followed by a low power phase with provocation in which the participant (low power) is confronted with the unfair other’s competitive economic choices (i.e., high gain for himself and low gain for the participant) and derogatory feedback messages (e.g., “You are annoying”). Conversely, in this low power phase, the fair other chooses cooperative economic choices (i.e., high gain for himself and the participant) and nice feedback messages (e.g., “You are very nice”). In line with previous literature^3,4,14,21^, this confrontation with the unfair as compared to the fair other during the low power phase serves to provoke anger in participants. Finally, participants engage in a high power phase after provocation in which their behavioral choices after anger provocation are measured. To isolate the impact of the players’ intentional choices from high or low monetary outcomes per se, control conditions are included in which there are only high or low gains for both players to choose from.

In addition to measuring participants’ brain activation by means of fMRI, we assessed the effects of the unfair other’s behavior on participants’ feelings through questionnaire-based measures of participants’ emotions (see Methods). The extent to which participants engaged in cooperative as opposed to competitive economic choices towards the unfair other in the high power phase following anger provocation (compared to baseline) served to assess the degree of participants’ punishment inhibition.

Overall, our study had three main objectives. First, to test whether the behavioral patterns and feelings evoked by the IG^2^ can be replicated in a neuroimaging study. Second, to validate the IG with neuroimaging data by investigating the brain activations related to the different events and phases of the IG. Thirdly, we aimed at delineating brain activations related to anger, aggression and punishment inhibition.

## Results

### Replication of behavioral patterns in the Inequality Game

**Figure 2:**
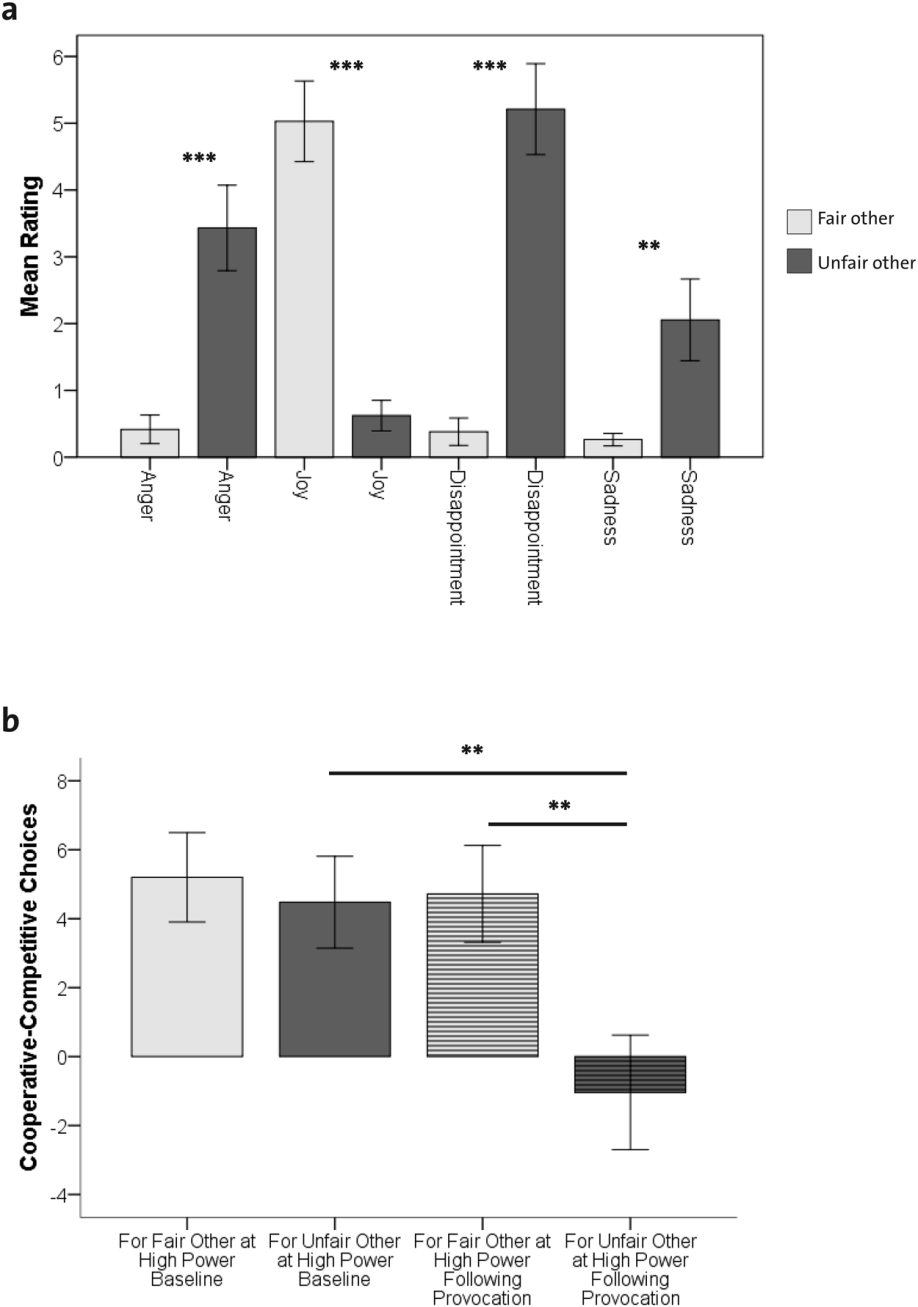
Effects of anger provocation in the low power phase on self-reported emotions and behavior. (**a**) Self-reported feelings in response to the fair other’s cooperative choice and the unfair other’s competitive choice in the low power phase. Bars depict means and + /−1 standard error. Asterisks refer to follow-up comparisons with **P < 0.01; ***P < 0.001. (**b**) Participants’ economic behavior towards the fair and unfair other in the high power phase at baseline and the high power phase following provocation. Bars depict means and + /−1 standard error. Asterisks show the results of follow-up pairwise comparisons with **P < 0.01.

In the current study, the behavioral characteristics of the IG^2^ could be replicated in a sample of participants with low scores of trait aggression as assessed by questionnaires (for details, see Supplementary Table 1) who on average did not engage in aggressive behavior towards the other players (i.e. competitive decisions) during the high power baseline phase of the IG (Fig. 2 and Supplementary Tables 1–5 and Supplementary Figs S1–4 in Part A–Behavioral Data).

To investigate participants’ feelings in response to the confrontation with a fair versus an unfair other in the IG, questionnaire-based data were submitted to a repeated-measure MANOVA with the within-subject factor *other* (2 levels: fair and unfair) and the self-reported feelings of anger, joy, disappointment, and sadness in response to the other’s economic choices as dependent variables. Results revealed a main effect of *other* across all emotions (*F*(4,21) = 13.75, *P* < 0.001, *ƞ²* = 0.72) As depicted in Fig. 2a, follow-up tests confirmed previous behavioral results^2^ by showing that exposure to competitive choices of the unfair other player during the low power phase reliably evoked feelings of anger, disappointment, and sadness, whereas exposure to cooperative choices by the fair other reliably induced feelings of joy. The same pattern of feelings (more anger, disappointment and sadness and less joy) was observed in response to derogatory as opposed to nice feedback as well as towards the unfair as opposed to the fair other player as a person (Supplementary Figs S1 and S2, respectively).

Behavioral decisions were analyzed with a 2 × 2 repeated-measure ANOVA with the within-subject factors *other* (fair and unfair) and *phase* (high power at baseline and high power after provocation), using the dependent variable *economic choice (amount of cooperative minus competitive choices for the other player)*. This revealed a significant main effect of *other* (*F(1,24)* = *16.46*, *P* < 0.001; *ƞ²* = 0.41) and *phase* (*F(1,24)* = *12.62*, *P* < 0.01; *ƞ²* = 0.35), as well as an interaction between *other* and *phase* (*F(1,24)* = *7.44*, *P* < 0.05; *ƞ²* = 0.24). As depicted in Fig. 2b follow-up comparisons showed that while economic choices towards the fair and unfair other were mainly cooperative at baseline and did not differ, participants displayed more punishment towards the unfair other after provocation (*P* < 0.01), while there were no changes in participants’ behavior towards the fair other player. As depicted in Supplementary Fig. S3, the same pattern of behavior was observed for feedback messages (nice, derogatory) sent to the other. These data indicate that exposure to unfair behavior did not only evoke feelings of anger (and reduced joy), but also promoted more punishment behavior toward the unfair other.

In spite of this effect, binomial tests revealed that participants’ cooperative economic choices towards both other players in the high power phase at baseline were significantly more frequent than expected by chance distribution (*P* < 0.001) and that in spite the low power phase with provocation there was an overall tendency for prosocial behavior to be maintained during the high power phase after provocation (*P* = 0.05, Supplementary Table 5).

Interestingly, self-reported feelings of anger towards the unfair other predicted the change in punishment behavior towards the unfair other from the first high-power phase at baseline to the subsequent high-power phase after provocation (*r* = 0.54, *P* = 0.005). In line with previous findings^2^, we also found that empathic concern was positively correlated with the extent to which participants inhibited their aggressive behavior towards the unfair other in the high power phase after provocation (*rs* = 0.41, *P* < 0.05), i.e., the frequency of cooperative rather than competitive economic decisions in this phase. A similar trend was observed for perspective taking (*rs* = 0.37, *P* = 0.07).

### Validation of the Inequality Game with neuroimaging data

As a first manipulation check of our neuroimaging data, we examined brain activation patterns evoked when, across all phases, participants saw the face of another player at the start of a given trial (Supplementary Table 6 in Part B–Neuroimaging Data). This revealed significant increases in occipital cortex and fusiform gyrus, two brain regions typically engaged during visual processing and face perception^22,23^, respectively.

As a second manipulation check, we tested which brain activity was elicited during economic choices or the selection of feedback messages across all phases. As detailed in Supplementary Table 7 in Part B–Neuroimaging Data, activations were increased in the precuneus, DLPFC, ACC and mid cingulate cortex (MCC; *P* < 0.05 FWE corrected). The DLPFC is a key area for attention and action selection^24^, while ACC and MCC play an important role in response conflict monitoring^25^ and social decision making^26,27^. Together with the precuneus, which has rich connections to other brain areas involved in sensorimotor, memory, and cognitive processing^28^, this distributed activation pattern suggests an integration of decision-making processes with motor planning and self-monitoring processes.

As a third step of validating the IG on a neuroimaging level, we tested whether, during the low power phase with provocation, cooperative choices of the fair other would elicit different brain activations than competitive choices of the unfair other. When participants were confronted with competitive choices of the unfair other (as opposed to the control condition with the same monetary outcome), increased activations in the inferior occipital cortex were measured (Supplementary Table 8, *P* < 0.05 FWE corrected), presumably due to enhanced visual attention to the displayed outcome. Conversely, cooperative choices of the fair other (as opposed to the control condition with the same monetary outcome) were associated with increased activations in distributed areas spanning the temporal cortex, frontopolar cortex, posterior cingulate cortex (PCC), and the ventral striatum (Supplementary Table 8, *P* < 0.05 FWE corrected). This suggests an interaction between brain networks implicated in social cognition^29^ and the ventral striatum that is crucial for reward processing in general^30^ and social reward processes in particular^31,32^.

### Brain activity related to anger, aggression and punishment inhibition

To test the brain activations elicited by being confronted with an unfair other, we contrasted BOLD activations when participants saw the face of the unfair versus the fair other player in the low power phase with provocation. This revealed that seeing the unfair as opposed to the fair other’s face produced significant activations in somatosensory cortices (Supplementary Table 9, *P* < 0.05 FWE corrected). No other significant activations were observed when contrasting fair and unfair faces in each of the phases of the IG (Supplementary Table 9).

In a next step, we investigated in how far neural responses to an unfair other in the low power phase with provocation were related to feelings of anger and to subsequent aggression and punishment inhibition.

**Figure 3:**
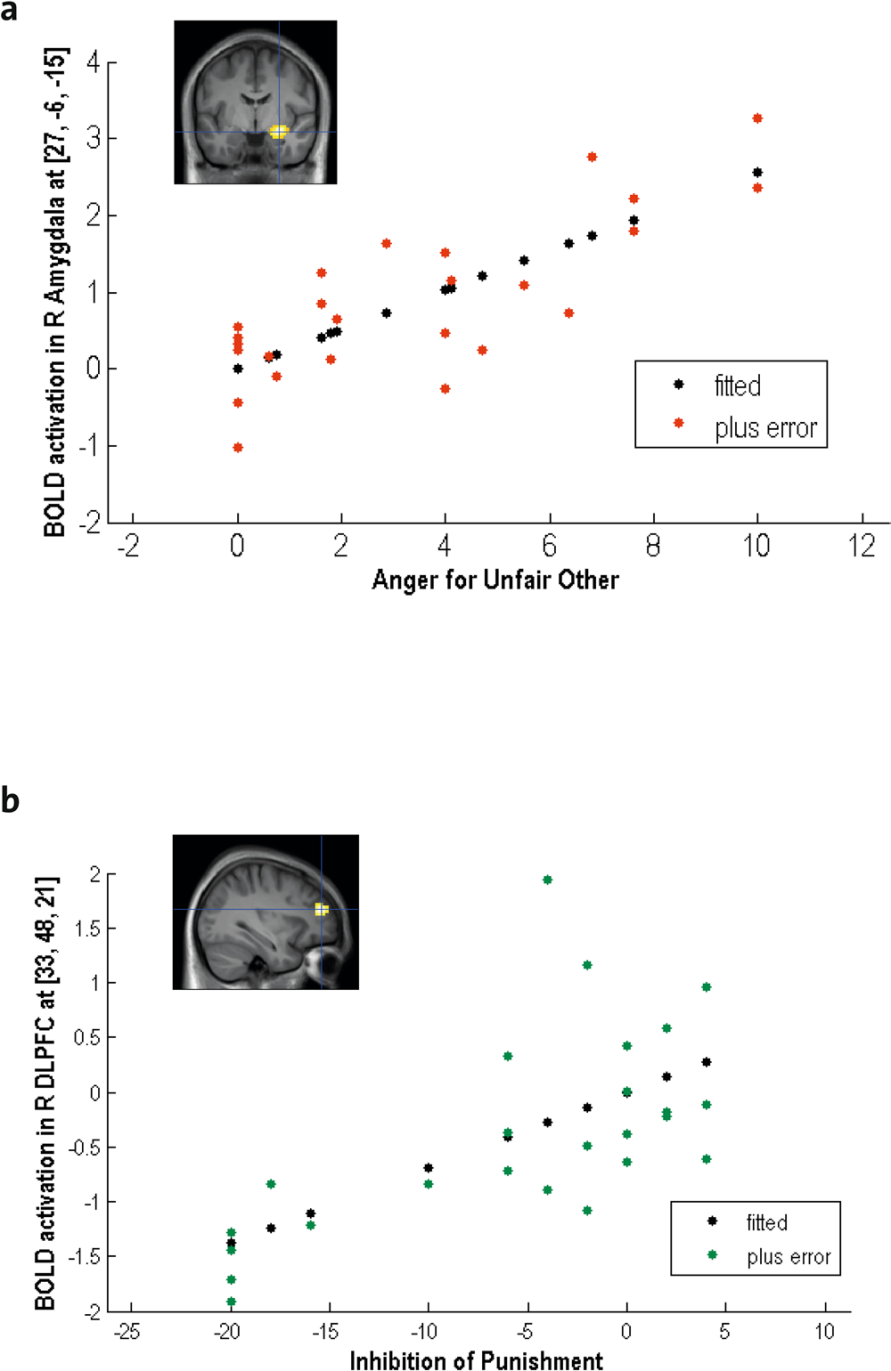
(**a**) Activations in right amygdala when seeing the unfair other’s face during the low power phase with provocation are parametrically modulated by self-reported feelings of anger. **(b)** Activations in bilateral dorsolateral prefrontal cortex (DLPFC) when seeing the face of the unfair other during the low power phase predicted the inhibition of punishment behavior during the subsequent high power phase. R, right.

First, we tested how brain activations when seeing the unfair versus fair other were correlated to self-reported feelings of anger towards the unfair other. This revealed that self-reported anger was associated with higher activation in this contrast in the amygdala, superior temporal sulcus (STS), and fusiform gyrus (Fig. 3a, Supplementary Table 9, *P* < 0.05 FWE corrected). These brain areas are involved in processing social information^29^, faces^22,23^, and affectively relevant cues^33,34^. Interestingly, higher activity in bilateral anterior DLPFC and ACC – two interconnected regions^24^ that are important for emotion regulation^35^ – in the same contrast predicted more frequent inhibition of subsequent punishment behavior by the participant (Fig. 3b, Supplementary Table 9, *P* < 0.05 FWE corrected). This effect was specific for the low power phase. In fact, there was no correlation between brain activations when seeing the unfair versus fair other’s face and the inhibition of aggressive behavior after provocation during the high power phase at baseline or the high power phase after provocation (for details, see Supplementary Table 9).

**Figure 4:**
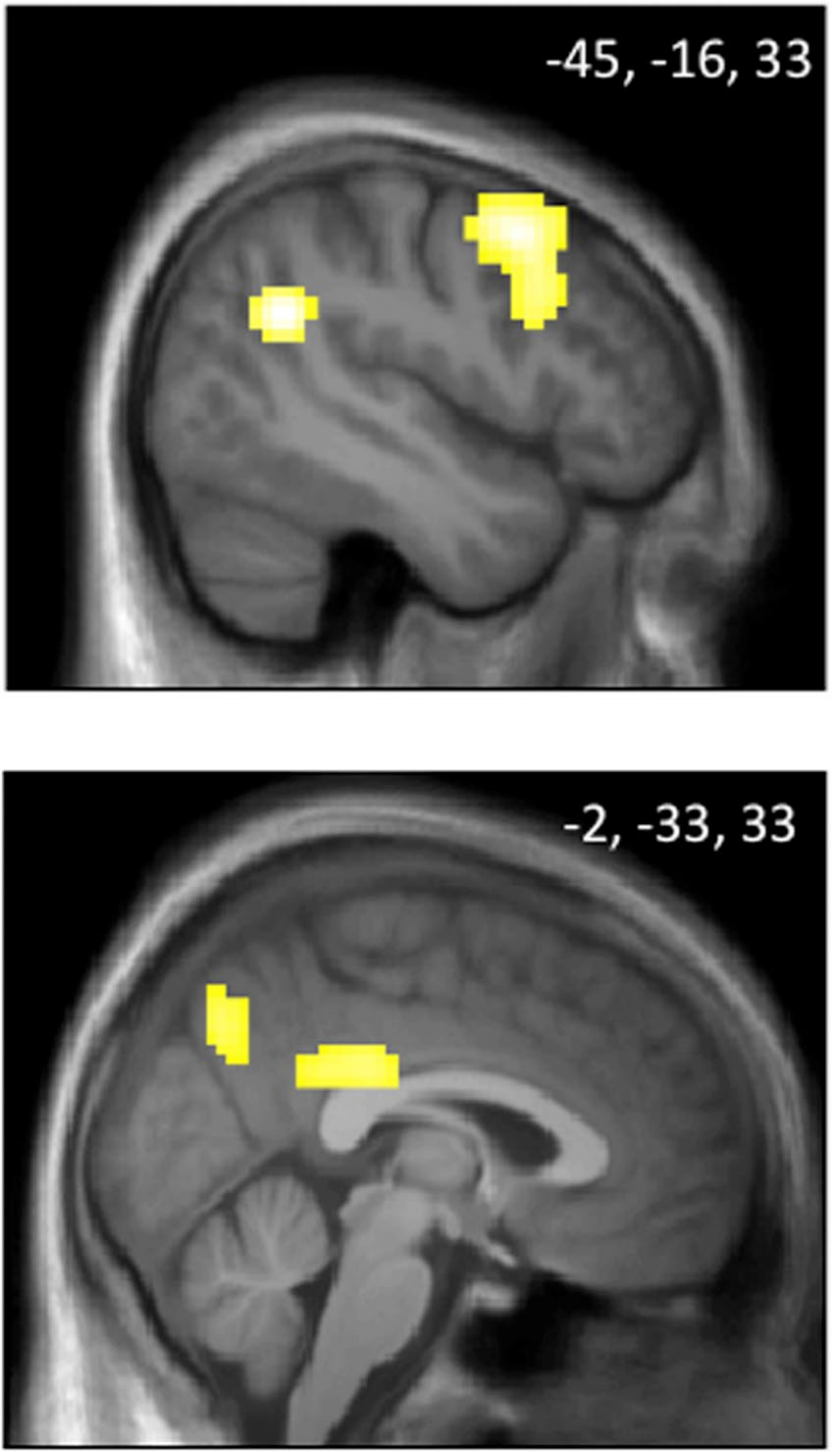
Activations in superior temporal gyrus, dorsolateral prefrontal cortex, posterior cingulate cortex and precuneus when participants make relevant economic choices for the unfair other as opposed to the fair other in the high power phase after provocation minus the high power phase at baseline (for details, see Supplementary Table 10). Numbers indicate MNI coordinates of the shown slices.

In order to test which brain activations are involved in the actual behavioral choices for the unfair as opposed to the fair other, we compared conditions when participants made active economic choices (as opposed to control choices) for the unfair as opposed to the fair other in the high power phase after provocation. This comparison revealed higher activations in several areas involved in social cognition^29^ and behavioral adjustment^36^, comprising PCC, precuneus and middle temporal gyrus (Supplementary Table 10, *P* < 0.05 FWE corrected). As depicted in Fig. 4, higher involvement of PCC, precuneus and superior temporal gyrus were also present when testing the interaction of the factors *phase* and *other* by comparing choices made for the unfair versus the fair other in the high power phase after provocation, relative to the high power phase at baseline (Supplementary Table 10, *P* < 0.05 FWE corrected). In addition, this contrast also revealed activations in the DLPFC (Supplementary Table 10, *P* < 0.05 FWE corrected). This finding suggests that when participants make choices for the unfair as opposed to the fair other player in the high power phase after provocation, DLPFC activations important for emotion regulation^35^ still play a role together with activations related to social cognition^29^ and behavioral adjustment^36^ encompassing the PCC, precuneus and temporal gyrus. The specificity of these results was confirmed by showing that no other contrast exhibited these activation patterns (for details, see Supplementary Table 10).

In an exploratory analysis, we wanted to test to what degree the brain activations in bilateral DLPFC in response to the face of the unfair other during the low power phase with provocation that were predicitive of later punishment inhibition were linked with brain activations during the actual economic choices for the unfair other in the high power phase after provocation. To this end, we carried out the following analysis: Using marsbar, we extracted activations from a 9 mm sphere around the peaks of the R and L DLPFC from the regression of the contrast *Unfair – Fair Other’s Face in low power phase with provocation* with the inhibition of subsequent punishment behavior (from Supplementary Table 9). In a next step, these values were entered in a regression analysis with the contrast of participants’ economic choices for the unfair versus fair other in the high power phase after provocation (from Supplementary Table 10). As detailed in Supplementary Table 10, this regression revealed that the R DLPFC activations when seeing the unfair other during the low power phase with provocation that were related to the later inhibition of punishment correlated with activations in the left primary and supplementary motor cortex when participants actually made their choices for the unfair other (*P* < 0.05, FWE corrected, cluster level threshold). In other words, brain activations related to emotion regulation^35^ during the provocation phase are positively correlated with activations in motor cortices of the contralateral side to the hand with which participants indicated their decisions for the unfair other in the high power phase after provocation, suggesting a long-term link between processes related to emotion regulation during provocation and later action selection. Supporting the consistency of this finding, an equivalent relation was found for L DLPFC activations, although these activations were only marginally significant with *P* = 0.06 (FWE corrected, cluster level threshold).

## Discussion

In the present study we do not only replicate the validity of the IG to elicit anger and punishment behavior in controlled settings, but also extend it to the neuroimaging level by highlighting specific neural substrates for emotional and behavioral processes recruited in these conditions. Specifically, we unveil dissociable networks related to feelings of anger and the control of subsequent punishment, implicating brain activations involved in social cognition and inhibitory control, respectively. We also report that social decision making differentially involves a distributed network including the PCC, precuneus, and superior temporal gyrus when directed to an unfair (versus fair) other.

On the behavioral level, this study shows for the first time that participants engage in cooperative economic choices for both other players in the high power phase at baseline. At the same time, the present data confirm previous behavioral findings^2^ by showing an increase in punishment behavior for the unfair other player in the high power phase after provocation. This indicates that punishment behavior in the IG is a result of the unfair other player’s behavior during the low power phase with provocation. The current study also confirms previous findings on the level of elicited emotions^2^ by showing that the confrontation with the unfair other’s competitive economic behavior and derogatory feedback also induces anger, disappointment and sadness, while reducing feelings of joy. In addition, we replicate that the higher participants score on empathic concern, the more they inhibit punishment behavior after provocation^2^.

On the level of brain imaging, previous studies on anger have often employed paradigms that did not necessarily induce true anger in the participant, such as the presentation of angry faces^9,10^, recall of anger-eliciting events^11^, imagery^12,13^, or rumination^14^. Moreover, these paradigms yielded very divergent findings, which might be due to the indirect and less reliable method of inducing anger.

In the present study, we found that the intensity of experienced anger when seeing the face of the unfair other was parametrically related to activations in amygdala, STS, and fusiform gyrus. While these findings differ from previous results obtained with paradigms indirectly studying anger^10,11^, they are in line with script-based studies^12,13^ on the implication of the STS in anger experience. Activations in the amygdala and STS have also been observed to be parametrically modulated by the intensity of a painful stimulus that was administered to an unfair other in a study in which anger and aggression were alternating^7^. The present study goes beyond the past studies in showing anger-related activations in the amygdala – a crucial component of emotion networks which is linked to affective appraisals and elicitation of defensive behaviors in animals^16^. In humans, amygdala activity signals motivational relevance^33,34^, whether for positive or negative events^35,37^, and promotes learning of associations with rewards or punishments^38^. Together with temporal areas involved in social cognition and mentalizing^29^, and activations in the fusiform gyrus, which is important for face processing^22,23^, these activation patterns may reflect appraisal and learning mechanisms related to provocation, yielding subjective feelings of anger.

Interestingly, unlike activity in the latter affective and social network, stronger activations in the DLPFC and ACC when seeing the unfair other during the provocation phase predicted the inhibition of aggressive behavior in the subsequent economic interaction phase. These two functionally interconnected regions^24^ are important for conflict resolution^24^, emotion regulation^18,35^, and the integration of motivational information for guiding goal-relevant decisions^24,39^. DLPFC lesions in animals and humans have been linked to increased aggression^17^ and transient DLPFC inhibition by transcranial magnetic stimulation was shown to reduce fairness behavior^40^. Previous studies also pointed to DLPFC involvement in third-party punishment^41^ or norm enforcement in bilateral economic interactions with responsible agents^40^. Moreover, impairments in the DLPFC and ACC have been reported for antisocial populations^20^. Their engagement in our paradigm might reflect how aversive experiences with an unfair other are integrated with contextual factors to determine future behavioral intentions, including punishment and the inhibition thereof.

In the economic interaction phase, greater activation was observed in PCC, precuneus and middle temporal gyrus when participants made behavioral decisions directed to the unfair other (as compared to the fair other). This pattern converges with previous observations during social decision making with provocative others^8^. The precuneus has rich connections to other brain areas involved in processing sensori-motor, visual, and cognitive information^28^. Together with activations in the PCC, which has been proposed to play a key role in contextual memory, change detection and subsequent behavioral adjustments^36^, and temporal lobe areas implicated in social cognition^29^, this activation pattern may be indicative of deeper integration of social and contextual information in decision making for unfair others.

In line with this observation, participants’ economic choices for the unfair as opposed to the fair other in the high power phase after provocation compared to the high power phase at baseline also showed increased activations in PCC, precuneus and temporal gyrus. In addition, this contrast revealed activations in the DLPFC, which is a key region for emotion regulation^35^. This brain activation pattern may represent the integration of emotion regulation with social decision-making during the inhibition of punishment behavior. In line with our previous study^2^ the behavioral data of the present study show that although participants on average punish the unfair other more than the fair other (Fig. 2b), the majority of participants can be classified as prosocial (Supplementary Table 5). In other words, even in the high power phase after provocation, patricipants select predominantly cooperative economic outcomes for the fair and the unfair other. In order to implement such an inhibition of punishment, the observed co-activations of regions involved in behavioral decisions, social cognition and emotion regulation may play a crucial role. To test for the robustness of this finding, future research is needed to investigate the role of these brain activations in more detail and with larger sample sizes and a variety of paradigms.

In summary, we extend previous work by dissociating the brain activity related to anger experience during provocation from subsequent regulations of punishment behavior. Anger-related brain activations when seeing an unfair other person were present in amygdala and STS, suggesting an interaction of temporal areas involved in social cognition^29^ with amygdala activations related to relevance and emotion processing. Stronger activations in DLPFC and ACC when seeing the unfair other predicted less punishment later on, emphasizing the importance of emotion control during the provocation phase in guiding subsequent punishment inhibition. As these networks were previously linked to impaired aggression regulation after brain lesions^17^ and in antisocial populations^20^, our findings provide new insights on functional mechanisms of aggression regulation that have important implications for effective clinical interventions in these populations. More generally, our paradigm may provide a novel and ecological tool to explore social and emotional processing in neuropsychiatric conditions associated with altered control of anger and aggressive behaviors.

## Methods

### Participants

We tested 32 male participants, of whom 7 had to be excluded as they did not believe that they were interacting with other players during the IG. The final sample thus comprised 25 men (mean age = 26.08 years, *SD* = 4.51). 13 of the participants were students (no students from psychology), 10 employees, and 2 had other jobs. We only tested male volunteers, due to previously reported sex differences in aggression^42,43^ and in emotional as well as neural responses to unfairness^44^. In addition to being male, volunteers had to be right-handed, aged 18–40, and without contraindication for magnetic resonance imaging (MRI). Further inclusion criteria comprised an alexithymia score <61 (using the Toronto- Alexithymia Scale, TAS-20^45^), as well as a depression score <29 (using Beck’s Depression Inventory, BDI-II^46^). The study protocol was approved by the Research Ethics Committee of the Faculty of Psychology and Educational Sciences at the University of Geneva. It was carried out in accordance with the approved guidelines and the declaration of Helsinki. Prior to the experiment participants provided informed written consent. They were paid and debriefed after the experiment. Materials and data related to this experiment will be made available upon request.

### Measures

#### Questionnaires

Prior to the experiment, we collected the following online questionnaires (using Questionnaire Machine, developed by Christoph Hofstetter, University of Geneva): the trait measures of the State-Trait Anger Expression Inventory^47,48^, the Aggression Questionnaire^49,50^, the Levenson Self-Report Psychopathy Scale^51,52^, the Behavioral Inhibition System/Behavioral Activation System Scales^53,54^, the trait form of the State-Trait Anxiety Inventory^55,56^, and the Interpersonal Reactivity Index^57,58^. The psychometric characteristics of the sample are summarized in Supplementary Table 1. In line with our previous behavioral work using the Inequality Game, IG^2^, we assessed participants’ feelings in relation to the task. In the current study, we used several self-report questionnaires with analogue response scales that ranged between 0 (not at all) to 10 (extremely). After the fMRI measurement, participants filled in these questionnaires to characterize their general feelings towards both other players as well as feelings evoked by the different types of interactions that took place during the three phases of the IG. Participants also evaluated how fair, agreeable, reliable, and good-looking they considered each of the other players, and finally provided information about their subjective involvement in the game.

#### Inequality Game

We acquired fMRI data while participants completed the IG^2^. The IG is an interactive game with economic choices and feedback messages in which the participant is sequentially paired with one of two other players (a fair and an unfair other who are counterbalanced across participants). Unbeknownst to the participant, the other players’ behavior is preprogrammed. As depicted in Fig. 1a the employed version of the IG consists of a high power phase at baseline to assess participants’ spontaneous behavior towards the other players prior to provocation, a low power phase with provocation in which anger is elicited and a subsequent high power phase after provocation in which punishment behavior is measured. Inspired by previous work^3,4,14,21^, the IG induces anger by confronting the participant with an unfair other player who engages in competitive economic choices (e.g., a high gain of 9 or 10 CHF for himself and a low gain of 0 or 1 CHF for the participant) and sends derogatory feedback messages (e.g., “You are annoying”) as opposed to a fair other player who makes cooperative economic choices (e.g., a high gain of 9 or 10 CHF for both, himself and the participant) and sends nice feedback messages (e.g., “You are very nice”). This is done in a low power phase with provocation. In a subsequent high power phase after provocation, participants’ behavioral reactions to the other players are assessed by measuring the degree to which participants choose competitive as opposed to cooperative economic distributions and derogatory as opposed to nice feedback messages.

More specifically (see also Fig. 1), the current paradigm has a 3 × 2 × 2 within-subject design with the factors *power* (high power phase at baseline, low power phase with provocation, and high power phase after provocation), *other* (fair and unfair), and *event type* (economic choices and feedback messages). *Power* is manipulated in each phase and serves to (i) assess participants spontaneous behavioral choices for the two other players during the high power phase at baseline, (ii) induce anger by confronting the participant with the unfair other players’ behavior in the low power phase with provocation, (iii) assess participants’ behavioral responses to the provocation in the subsequent high power phase. The manipulation of *other* means that the participant is paired with either the fair or unfair other player during half of the trials. In line with the validated version of the IG^2^, we implemented two *event types:* joint economic choices on a 2 by 2 payoff matrix and a choice of one out of four feedback messages. To optimize time for fMRI measurements and maximize repetitions of economic interaction trials, the current fMRI version of the IG focuses on economic interactions (36 economic interactions per phase) and only uses 6 feedback trials per phase. In each phase (Fig. 1b), there are 20 interactions with *relevant economic choices*. In these *relevant economic choices* the person in high power can choose between outcomes that are competitive or cooperative. This means that the player with high power is in control of up to 90% of the other’s gain, while the player with low power can only affect up to 10% of the other’s gain. In addition, only the player with high power selects feedback messages. In each of these rounds, the player with high power controls the lines and starts by choosing between a cooperative (e.g., 9 or 10 CHF for both) or competitive outcomes (e.g., 9 or 10 CHF for himself and 0 or 1 CHF for the other). The player with low power chooses next and can only impact up to 1 CHF of the outcome (e.g., by choosing between 10 or 9 CHF for the other and 1 or 0 CHF for himself). The screens that participants see when they are in the high power condition are detailed in Fig. 1b. In order to control for monetary reward per se, 16 economic interactions in each phase have essentially pre-determined monetary outcomes for both players (half of them being “win” trials with high monetary outcomes for both players and half of them being “no win” trials with low or no monetary gains for both players). Participants were told that at the end of the game, the computer will randomly choose two interactions which will be paid out, which implies a gain range between 0 and 20 CHF.

We obtained a measure of *punishment inhibition* by computing the difference in the number of trials in which participants made cooperative as opposed to competitive decisions towards the unfair other during the high power phase after provocation, relative to the high power phase at baseline: i.e., *punishment inhibition* = (cooperative-competitive decisions for the unfair other during the high power phase after provocation) - (cooperative-competitive decisions for the unfair other during the high power phase at baseline). Feedback behavior, which was not of primary interest in the current study, was quantified by computing the sum of weighted frequencies with which participants chose the following four feedback sentences for each player: “You are very nice” was assigned a weight of 2, “You are nice” a weight of 1, “You are not nice” a weight of −1, and “You are annoying” a weight of −2. The feedback index thus indicates to what extent participants chose nice (positive values) or derogatory feedback (negative values) for each of the other players.

The Inequality Game can be seen as a variant of mixed-motive games, such as the Prisonner’s Dilemma Game^59^ to which a power manipulation is added^60^. However, in contrast to a Prisonner’s Dilemma game or other mixed-motive games, the payoffs of the two players interacting in in the IG are largely independent, thus disentangling self-related choices from other-related choices^2^. This means that the choice of high or low payoffs for the other player do not affect participants’ own payoffs.

#### Procedure

The experiment had a total duration of 2 h. Participants were first seated in a multi-computer room and told that during the fMRI scanning they would interact with two other participants who would arrive later. To increase credibility, we ostensibly prepared questionnaire sheets and pens for two other participants in the multi-computer room, to the right and left of the participants’ seat and told participants that the two other players would be seated there while participants were being installed in the MRI. Participants did not see the other players and unknown to the participants, the others´ choices in the IG were preprogrammed.

After explaining the general procedure of the experiment, participants filled in consent forms and questionnaires. The experimenter then took a photograph of the participant in order to allegedly display it to the others during the game. While the experimenter left the room under the pretense to upload the photograph, participants read the instructions of the game. Upon the experimenter’s return, their understanding of the game was tested by means of a questionnaire and oral probing. Participants were reminded that they could win between 0 and 20 CHF during the game in addition to the 35 CHF they obtained for participating in the experiment. Participants were then familiarized with the game through a few test trials allegedly played with the scanner operator who used the computer in the MRI control room. To underline the training purpose of test trials, letters replaced the economic outcomes and feedback sentences were numbered.

Following the preparation phase, participants were guided to the scanner room and brain fMRI data were obtained while they played the IG with the two ostensible other players in the multi-computer room. To increase credibility, the experimenter pretended to double-check whether all players were ready prior to starting the game. During the fMRI measurement, participants played the three phases of the IG (high power at baseline, low power with provocation, and high power after provocation). Each phase served as an fMRI run and lasted about 13 minutes. A structural brain scan (T1) was acquired at the end of the MRI measurement. Following the neuroimaging part, participants were guided to the waiting area of the laboratory where they filled in a few additional questionnaires about their overall feelings towards the other players, the emotions experienced in response to the different events of each phase of the game, and their thoughts on the experiment’s purpose. At the end of the experiment, participants received 45 CHF and a compact disc with their anatomical brain scan. They were then thanked, probed for suspicion, and debriefed.

#### fMRI

While participants played the IG, blood oxygenation level dependent contrast (BOLD) signals were acquired with a 3 Tesla Siemens Magnetom Trio Tim syngo (MR B17) scanner and a 32 channel head coil. 40 slices were obtained in descending order (TR = 2000 ms, TE = 20 ms; voxel size = 3 × 3 × 2.5 mm³). High resolution anatomical images (1 × 1 × 1 mm³) were recorded using a T1-weighted sagitally oriented GRAPPA sequence with 192 slices. Visual stimuli were presented on a back projection screen inside the scanner bore using an LCD projector (CP-SX1350, Hitachi, Japan).

#### Analyses

Statistical analyses of behavioral data were carried out using the software SPSS Statistics 21. Neuroimaging data were analyzed using the software SPM 8 (Wellcome Trust Centre for Neuroimaging, London) run on Matlab R2012b. Imaging data were preprocessed with the following steps: first, all structural and functional data were manually reoriented, so that the anterior commissure was at the origin of the coordinate system (0, 0, 0). Then, data were realigned and coregistered using standard procedures. Warping parameters extracted from the segmentation of the anatomical images were used to normalize functional and structural images to the Montreal Neurological Institute (MNI) template brain. Functional images were resampled to a 3 mm³ voxel size and spatially smoothed using a Gaussian kernel of 6 mm³ full-width at half-maximum (FWHM).

First-level general linear models for each participant were computed in a single design matrix using each of the three phases as a run (high power at baseline, low power with provocation, high power after provocation). Regressors were modeled in an event-related design according to the display frames (Fig. 1). For each high power phase, the onsets and durations of the following regressors (events) were separately modeled for interactions with the fair and unfair other (including control conditions with pre-determined “win” or “no win” outcomes): display of the other’s face, payoff display, participant’s decision, display of the participant’s own decision, and display of the other’s decision (which served at the same time as the final result display). In addition, we included participants’ decisions for feedback choices and the related feedback presentation in the model. These conditions were not of interest for the current analysis, because there were too few observations (only 3 feedback interactions with each of the other players per phase, see above). Corresponding regressors were modelled for events in the low power phase. Movement parameters from each run were included as additional regressors.

On the second (group) level, we carried out general linear models on the first level contrast images, after smoothing by a Gaussian kernel of 9 mm³ FWHM. Statistical comparisons between conditions were performed by standard whole-brain analyses with one-sample *t*-tests, analyses of variance, and linear regressions in SPM. Significance levels were set to *P* < 0.05, FWE corrected at the voxel or cluster level. In order to obtain robust statistics^61,62^, while at the same time avoiding the risk of missing true effects (which can be much smaller in affective and social neuroscience than in motor or perceptual research^63^), we have adopted the following strategy: To test for basic effects related to perceptual or motor phenomena, we use a very conservative threshold of P < 0.05 FWE correction at the voxel level. To study more subtle effects related to affective and social phenomena, we employ a cluster level threshold with a FWE correction of P < 0.05. Note that this thresholding is more conservative than the thresholding of P < 0.005 with a 10 voxel extent that has been suggested for neuroimaging studies of affective and social phenomena^63^.

## Electronic supplementary material

Supplementary Information
