# Supplementary material for: Distinct Brain Areas involved in Anger versus Punishment during Social Interactions
Source: Sci Rep. 2018 Jul 12;8:10556. doi: 10.1038/s41598-018-28863-3 (PMC6043598; doi:10.1038/s41598-018-28863-3)
Supplement: Supplementary file 1 — Supplementary Information [file 41598_2018_28863_MOESM1_ESM.pdf]

Supplementary Information

Distinct Brain Areas involved in Anger versus Punishment during Social Interactions

Olga M. Klimecki[1,2,3], David Sander[1,2], Patrik Vuilleumier[1,3]

[1] Swiss Centre for Affective Sciences, University of Geneva, Chemin des Mines 9, 1202 Geneva, Switzerland.

[2] Laboratory for the Study of Emotion Elicitation and Expression, Department of Psychology, University of Geneva, Boulevard du Pont d' Arve 40, 1205 Geneva, Switzerland.

[3] Laboratory for Behavioral Neurology and Imaging of Cognition, Department of Neuroscience, Medical School, University of Geneva, rue Michel Servet 1, 1211 Geneva, Switzerland.

SUPPLEMENTARY INFORMATION

PART A – Behavioral Data

Supplementary Table 1. Summary of individual psychometric data across all participants.

	Mean	*Standard Deviation*
Depression (BDI)	3.12	3.66
Alexythimia (TAS)	50.64	4.5
Trait Anger (STAXI)	19.08	5.38
Angry Temperament	6.16	1.6
Angry Reaction	9.6	3.63
Anger Expression/ Control (STAXI)	28.44	10.68
External Anger Expression	14.88	2.52
Internal Anger Expression	17.72	3.65
External Anger Control	26.64	5.07
Internal Anger Control	25.52	5.35
State Anxiety (STAI)	27.32	4.41
Aggression (AQ)	57.88	9.68
Physical Aggression	16.36	4.3
Verbal Aggression	14.12	2.73
Anger	13.28	2.54

Hostility	14.12	4.18
BAS Drive	12.68	2.06
BAS Fun Seeking	12	1.66
BAS Reward Responsiveness	17.88	1.54
BIS	18.44	3.68
Primary Psychopathy	29	7.35
Secondary Psychopathy	17.4	4.07
Empathy (IRI total)	68.76	12.12
Perspective Taking	19.64	4.44
Fantasy	19.24	5.23
Empathic Concern	20	5.63
Personal Distress	9.88	4.65

Note. AQ, Aggression Questionnaire; BAS, Behavioral Activation System; BDI, Beck's Depression Inventory; BIS, Behavioral Inhibition System; IRI, Interpersonal Reactivity Index; *M*, Mean; STAI , State-Trait Anxiety Inventory; STAXI, State-Trait Anger Expression Inventory; *SD*, Standard Deviation; TAS, Toronto- Alexithymia Scale. One sample *t*-tests comparing aggression scores in our sample to the norms reported by Buss and Perry[1] revealed that participants in our sample had comparatively low scores on total aggression ($t(24) = -10.29$, $P < .001$) .

Supplementary Table 2. Participants' self-reports with regard to the Inequality Game on post-scanning questionnaires.

Self-report	Mean	Standard Deviation
Felling of self-implication	7.13	2.67
Feeling of others' presence	5.49	2.55
Importance of economic gain	6.18	3.2

Note: All ratings on a scale from 0, not at all, to 10, extremely.

Supplementary Table 3. The fair other is evaluated more favorably in post scanning questionnaires than the unfair other. A repeated measures MANOVA (Multivariate Analysis of Variance) with the within-subject factor *other* (2 levels: fair or unfair) and evaluations of the other (as fair, agreeable, reliable, and good-looking) as dependent variables revealed a main effect of other ($F(4,21)=6.35$, $P<.01$, $\eta^2 = .55$). *P* values in table refer to follow-up paired comparisons. *SD*, standard deviation.

Evaluation	Fair Other	Unfair Other	
	Mean (SD)	***Mean (SD)***	***P***
Fair	6.23 (2.97)	3.26 (2.76)	< .01
Agreeable	6.63 (2.66)	2.86 (2.17)	< .001
Good-looking	3.62 (3.13)	2.54 (2.21)	< .05
Reliable	6.06 (3.3)	2.28 (2.26)	< .001

Supplementary Table 4. Spearman's *rho (rs)* correlations of self-reported anger in response to competitive choices by the unfair other with scores on standard anger and aggression questionnaires. STAXI, State-Trait Anxiety Inventory; AQ, Aggression Questionnaire.

**Questionnaire Measure**	**Anger**
STAXI Anger Trait	$rs = .46$; $P < .05$
STAXI Angry Temperament	$rs = .39$; $P = .056$
STAXI Angry Reaction	$rs = .43$; $P < .05$
STAXI Internal Anger Expression	$rs = .48$; $P < .05$
STAXI External Anger Control	$rs = -.47$; $P < .05$
STAXI Anger Expression	$rs = .54$; $P < .01$
AQ Physical Aggression	$rs = .42$; $P < .05$

Supplementary Table 5. **Classification of participants in subgroups according to their economic choices for others in the Inequality Game.**

Classification	High Power Phase at Baseline	High Power Phase After provocation
**Prosocial** (choosing more cooperative economic outcomes for the fair and unfair other)	18 (higher than expected by chance; *P* < .001)	11 (marginally higher than expected by chance; *P* = .05)
**Sanctioning** (reciprocating by choosing more cooperative economic outcomes for the fair other and more competitive outcomes for the unfair other)	2 (lower than expected by chance; *P* < .05)	8 (*n.s.*)
**Competitive towards both players** (choosing more competitive economic outcomes for the fair and unfair other)	4 (*n.s.*)	5 (*n.s.*)
Not falling into any of the three categories	1	1

Note: Classification was based on behavior in the two high power phases [see also **Figure 2b**]. Binomial tests were used to assess whether frequency of behaviors per category and phase deviated from chance distributions. *n.s.,* not significant.

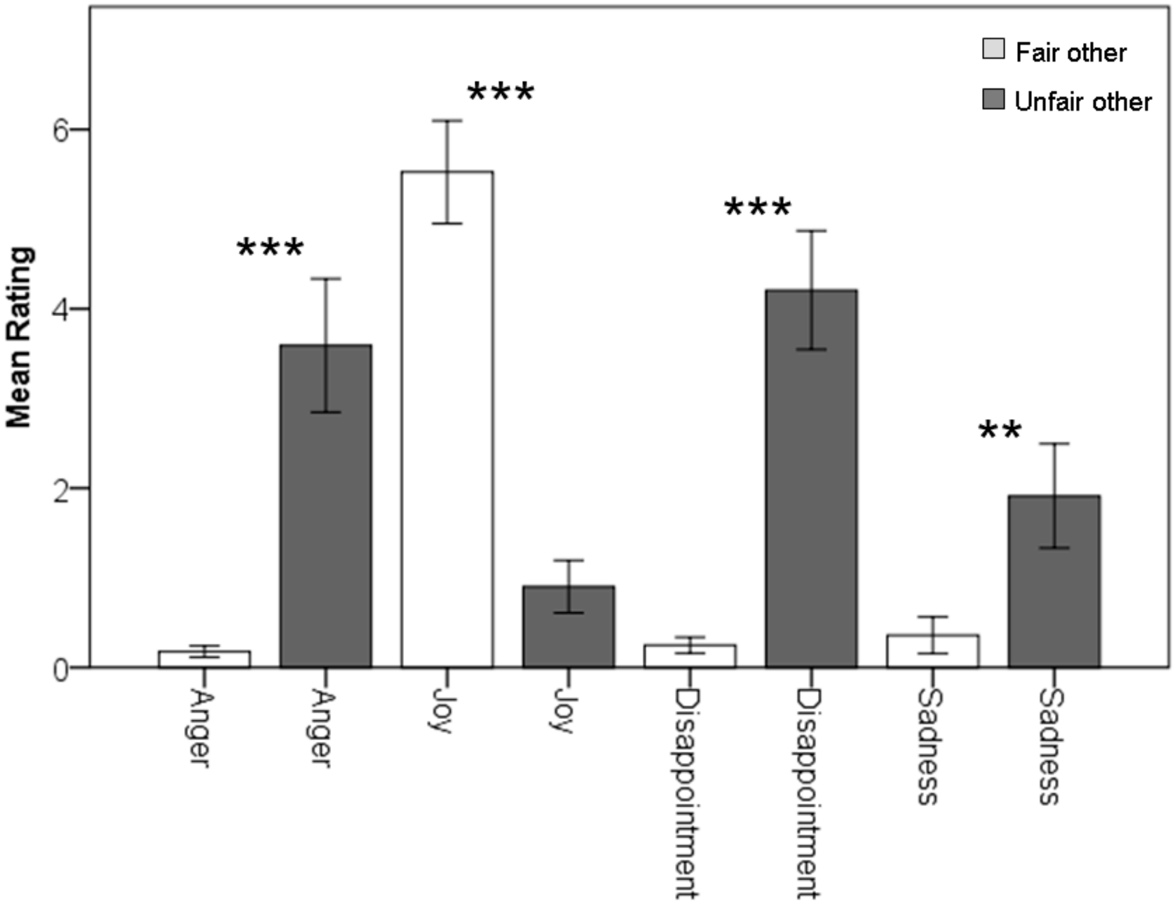

Supplementary Figure S1. Self-reported feelings in response to the fair other's nice feedback and the unfair other's derogatory feedback in the low power phase. A repeated measures MANOVA with the within-subject factor *other* (2 levels: fair and unfair) and the self-reported feelings anger, joy, disappointment, and sadness in response to feedback messages as dependent variables (anger, joy, disappointment and sadness) revealed a significant effect of *other* ($F(4,21) = 12.7$, $P < .001$, $\eta^2 = .71$). Bars depict means and +/- 1 standard error. Asterisks refer to follow-up comparisons with **, $P = .01$; ***, $P < .001$.

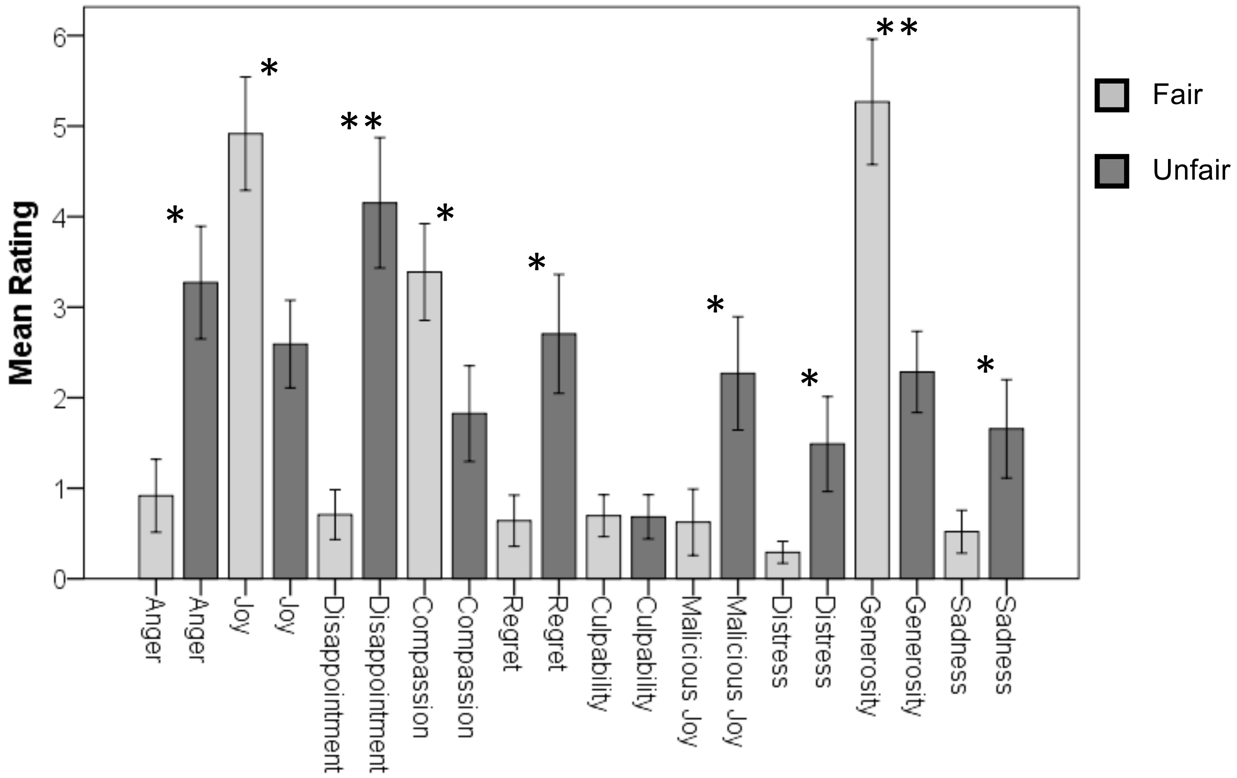

Supplementary Figure S2. **Self-reported feelings towards the fair and unfair other after the Inequality Game.** A repeated measures MANOVA with the within subject factor *other* (2 levels: fair and unfair) and the self-reported feelings of anger, joy, disappointment, compassion, regret, culpability, malicious joy, distress, generosity, and sadness towards the other player as dependent variables revealed a significant effect of *other* ($F(10,15) = 2.62$, $P < .05$, $\eta^2 = .64$). Bars depict means and +/- 1 standard error. Asterisks refer to follow-up comparisons with *, $P < .05$; **, $P < .01$.

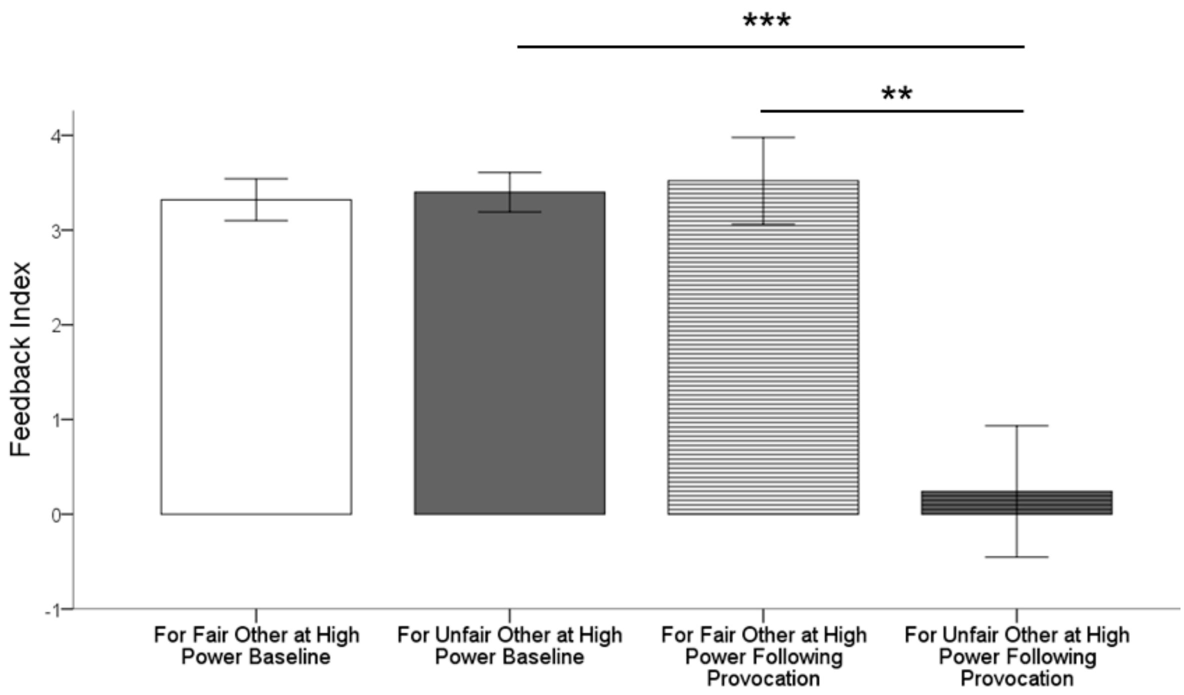

Supplementary Figure S3. Changes in participants' feedback behavior in the Inequality Game. A 2 x 2 repeated measures ANOVA with the within subject factors *other* (fair or unfair other) and *phase* (high power at baseline and high power after provocation) and the dependent variable feedback index revealed a significant main effect of *other* ($F(1,24) = 10.86$, $P < .01$; $\eta^2 = .31$) and *phase* ($F(1,24) = 13.45$, $P = .001$; $\eta^2 = .36$) and an interaction between *other* and *phase* ($F(1,24) = 13.24$, $P = .001$; $\eta^2 = .36$). Asterisks show the results of follow-up pairwise comparisons with **, $P < .01$; ***, $P < .001$. Bars depict means and +/- 1 standard error, the more positive the values on the y-axis, the nicer the feedback, negative values would indicate a prevalence of derogatory feedback.

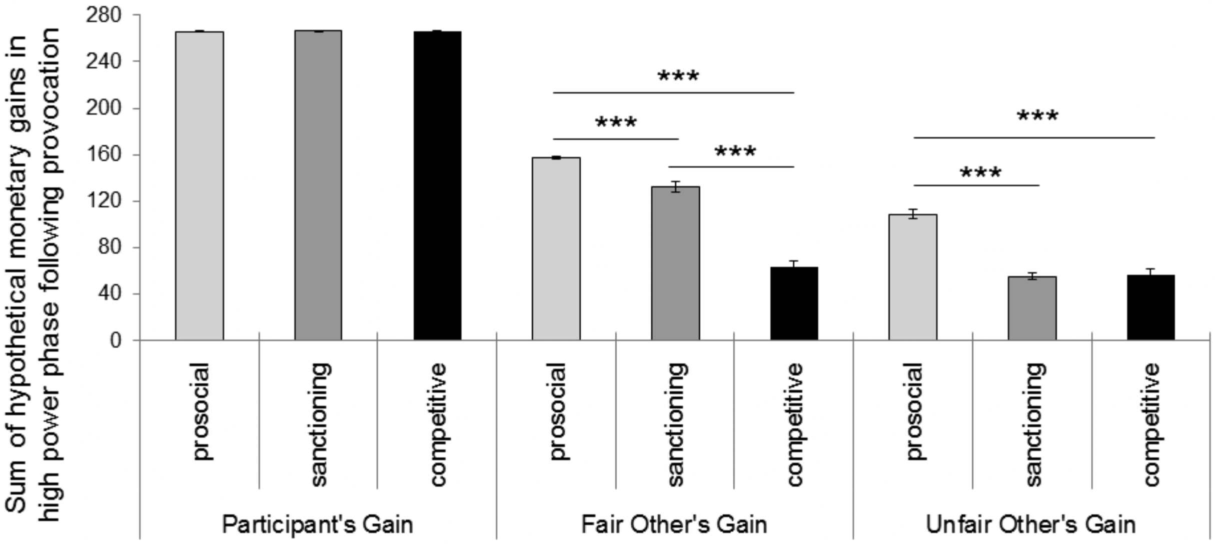

Supplementary Figure S4. Participants' classification is confirmed by differences in monetary gains. A MANOVA with the between subject factor *economic preference* (3 levels: prosocial, sanctioning, and competitive) and the three dependent variables *hypothetical overall gains for the participant, the fair other and the unfair other in the high power phase after provocation* showed an overall effect of economic preferences ($F(6,40) = 36.11$, $P < .001$, $\eta^2 = .84$). Univariate tests revealed that economic preferences had no effect on participants' own gains ($F(2,21) = 0.62$, $P = .55$), while the fair and unfair other's gains differed markedly (both $F(2,21) \geq 68.94$, $P \leq .001$), thus confirming the independence of the participant's gain from the others' gains. Bars depict means and two standard errors. Asterisks are related to follow-up pairwise comparisons with ***, $P < .001$. See also **Supplementary Table 5**.

PART B – Neuroimaging Data

Supplementary Table 6. First Manipulation Check: BOLD activation in response to the others' faces (across all phases).

Brain regions	Cluster size	MNI coordinates (mm)			*Z* values
		x	y	z	
L + R Occipital Gyrus & Fusiform Gyrus	2058	36	-66	-18	6.21
L Cerebellum		-33	-69	-15	6.11
R Cerebellum		39	-51	-21	5.93
L + R Frontopolar Cortex	201	0	57	-9	5.17
R Superior Orbital Gyrus		9	42	-12	4.99
L Superior Orbital Gyrus		-9	42	-18	4.70
L + R Middle Occipital Gyrus	46	-30	-84	18	4.96
L + R Precuneus	55	3	-60	27	4.83

Note. All values $P < 0.05$, FWE corrected (voxel level), $k \geq 10$ voxel. L, left; R, right; MNI coordinates and *Z* values are given for the grey matter peaks of the respective cluster.

Supplementary Table 7. Second Manipulation Check: BOLD activation related to active economic and feedback choices (across all phases).

Brain regions	Cluster size	MNI coordinates (mm)			*Z* values
		x	y	z	
L + R Precuneus	6712	15	-60	63	65535
L + R Cerebellum & Occipital Cortex		-36	-75	-24	7.03
L + R Inferior Parietal Cortex		33	-57	60	6.99
R Middle Frontal Gyrus	249	33	6	63	6.59
R Middle Orbital Cortex	236	42	51	-18	5.51
R Superior Orbital Cortex		24	42	-21	5.22
R Frontopolar Cortex		15	57	-18	4.65
L + R Medial Cingulate Cortex, ACC	182	3	21	39	5.44
R Posterior-medial Frontal Cortex		12	18	63	4.62
R Dorsolateral Prefrontal Cortex	178	45	39	21	5.37
L Superior Orbital Cortex	181	-42	48	-18	5.18
		-21	42	-21	4.81
		-9	45	-24	4.78
L Middle Frontal Gyrus	44	-48	12	48	4.93
L Cerebellum	10	-48	-60	-45	4.72

Note. All values $P < 0.05$, FWE corrected (voxel level), $k \geq 10$ voxel. MNI coordinates and *Z* values are given for the grey matter peaks of the respective cluster. ACC, anterior cingulate cortex; L, left; R, right.

Supplementary Table 8. **Low Power Phase: BOLD activation related to intentional cooperative or competitive economic choices made by the fair or unfair other, respectively, as opposed to an unintentional control condition with the same other player and the same monetary outcome for the participant.**

**Brain regions**	**Cluster size**	**MNI coordinates (mm)**			***Z* values**
		**x**	**y**	**z**	
Cooperative Choice of Fair Other *minus* Win with Fair Other					
L + R Thalamus	998	-9	-21	18	4.59
L Putamen		-21	-9	12	4.02
L + R Posterior Cingulate Cortex		3	-21	27	3.91
L Ventral Striatum/ Nucleus Accumbens		-9	6	-3	3.56
L + R Frontopolar Cortex	281	-3	63	-12	4.38
L Medial Temporal Pole	238	-45	15	-30	4.01
L Inferior Temporal Gyrus		-45	-6	-30	3.33
L Fusiform Gyrus		-36	-15	-27	3.25
Competitive Choice of Unfair Other *minus* No-Win with Unfair Other					
R Inferior Occipital Gyrus	474	42	-87	-6	4.52

Note. All values $P < 0.05$, FWE corrected (cluster level, cluster defining threshold $P < .001$). In this context, no significant activations were observed for the reverse contrasts or for relations with angry feelings or the inhibition of punishment behavior. MNI coordinates and *Z* values are given for the grey matter peaks of the respective cluster. L, left; R, right;

Supplementary Table 9. **BOLD activation in response to the unfair vs fair other's face, and correlations with self-reports and behavior.**

Brain regions	Cluster size	MNI coordinates (mm)			*Z* values
		x	y	z	
Unfair – Fair Other's Face in low power phase with provocation					
R Postcentral Gyrus (Somatosensory Cortex)	461	63	-12	42	4.7
		39	-12	69	3.17
Correlation with self-reported anger					
R Amygdala	326	27	-6	-15	4.72
R Middle Temporal Gyrus and STS		45	-27	-3	3.64
R Fusiform Gyrus		39	-42	-24	3.63
Correlation with inhibition of subsequent punishment behavior					
R Middle Frontal Gyrus (DLPFC)	677	33	48	21	3.45
L Middle Frontal Gyrus (DLPFC)		-33	36	33	3.45
L + R ACC		6	27	24	3.13

Note. Activations were computed using a factorial design with the within subject factors *phase* (high power at baseline, low power with provocation and high power after provocation). The first level contrasts pertained to the unfair versus the fair other's face. Correlations were computed with the contrast "unfair – fair face in the low power phase with provocation". Values are $P < 0.05$, FWE corrected (cluster level defining threshold $P < .001$) apart from the "Correlation with inhibition of subsequent punishment behavior", which is $P < 0.05$, FWE corrected (cluster level defining threshold $P < .005$). All other contrasts of the unfair versus fair face, including the reverse contrast fair minus unfair face yielded no significant brain activation. These tests include the high power phase at baseline, the low power

phase with provocation and the high power phase after provocation. No significant correlation between brain activation when seeing the unfair versus fair other's face with the inhibition of aggressive behavior after provocation was observed during the high power phase at baseline or the high power phase after provocation. Individual ratings of experienced anger in response to the unfair other's competitive choices were obtained in the post-scanning questionnaire session (see methods). Correlation with these ratings was assessed using a linear regression analysis across the whole brain. Inhibition of aggression was measured as the difference "cooperative-competitive choices" for the unfair other during the high power phase after provocation, minus the difference "cooperative-competitive choices" for the unfair other during the high power phase at baseline (see Methods). Correlation with this behavior was assessed using a linear regression analysis across the whole brain. In order to test whether for positive and negative functional connectivity of left or right DLPFC (which correlated with the inhibition of subsequent punishment behavior) with other brain activations, we extracted brain activity from a 9 mm sphere around the left and right DLPFC peaks using masrbar. We then correlated this activation with whole brain activity in the same contrast. Both, left and right DLPFC activations were correlated to ipsilateral and contralateral DLPFC activations and to ACC activations ($P < 0.05$, FWE corrected, voxel level). No negative correlations of the left and right DLPFC with other brain activations were observed.

MNI coordinates and Z values are given for the grey matter peaks of the respective cluster, ACC, Anterior Cingulate Cortex; DLPFC, Dorsolateral Prefrontal Cortex; STS, Superior Temporal Sulcus; L, left; R, right.

Supplementary Table 10. BOLD activation when making cooperative or competitive choices for the unfair versus fair other during the high power phase at baseline and the high power phase after provocation.

**Brain regions**	**Cluster size**	**MNI coordinates (mm)**			***Z* values**
		**x**	**y**	**z**	
<u>Choice for unfair other – fair other during the high power phase after provocation</u>					
L+R PCC	2151	-12	-54	27	5.04
L Angular Gyrus, L Middle Temporal Gyrus		-42	-54	27	4.5
L + R Precuneus		-3	-69	39	3.79
<u>Correlation of activations in R DLPFC activations when participants saw the unfair versus fair face of the other player in the low power phase with provocation that were correlated to punishment inhibition in the high power phase after provocation with BOLD activations during the Choice for unfair other – fair other during the high power phase after provocation</u>					
L presentral gyrus	904	-18	0	66	4.61
L supplementary motor cortex		-6	-3	54	4.08
<u>Interaction between phase and other: (Choice for unfair other – fair other during the high power phase after provocation) – (Choice for unfair other – fair other during the high power phase at baseline)</u>					
L Superior Temporal Gyrus	658	-45	-54	27	4.31
L PCC		9	-33	27	4.15

L Precuneus		-3	-69	39	3.62
L Middle Frontal Gyrus (DLPFC)	601	-42	9	48	4.43
		-39	6	51	4.23
L IFG (p. triangularis)		-42	15	36	3.79

Note. Activations were computed using a factorial design with the within subject factors *phase* (high power at baseline and high power after provocation) and *other* (fair and unfair other). The first level contrasts pertained to active economic choices (cooperative or competitive) versus control choices (win or no win trials). There were no main effects of phase ("choices for the fair and unfair other during the high power phase at baseline - the high power phase after provocation" and "choices for the fair and unfair other during the high power phase after provocation - the high power phase at baseline"). No activations were present for the contrast of choices for the "unfair - fair other" or "fair - unfair other" in the high power phase at baseline. Similarly, no significant activations were observed for the contrast "choices for the fair - unfair other in the high power phase after provocation". There were also no significant differences in brain activation for the economic choices for the unfair or fair other between the high power phases (i.e., for the contrasts "choice for unfair other during high power phase after provocation – choice for unfair other during high power phase at baseline" and "choice for fair other during high power phase after provocation – choice for fair other during high power phase at baseline"). Finally, there was no interaction effect between phase and other for the contrast "(choice for fair other during high power phase after provocation – choice for unfair other during high power phase after provocation) - (choice for fair other during high power phase at baseline – choice for unfair other during high power phase at baseline)". Values $P < 0.05$, FWE corrected (cluster level defining threshold $P < .001$). MNI coordinates and Z values are given for the grey matter peaks of the respective cluster. L, left; R, right, DLPFC, dorsolateral prefrontal cortex; PCC, posterior cingulate cortex; IFG, inferior frontal gyrus.

Supplementary References

1. Buss, A.H. & Perry, M. The Aggression Questionnaire. *Journal of Personality and Social Psychology* 63, 452-459 (1992).
